# Evaluation of Work Engagement and Its Determinants in Kermanshah Hospitals Staff in 2013

**DOI:** 10.5539/gjhs.v7n2p170

**Published:** 2014-10-17

**Authors:** Mohammad Mahboubi, Fariba Ghahramani, Mohsen Mohammadi, Nastaran Amani, Seyedeh Hoda Mousavi, Farida Moradi, Arash Akbarzadeh, Mahmmoud Kazemi

**Affiliations:** 1 Abadan School of Medical Sciences, Abadan, Iran; 2 Kermanshah University of Medical Sciences, Kermanshah, Iran; 3 School of Health, Shiraz University of Medical Sciences, Shiraz, Iran; 4 Student Research Committee, Kermanshah University of Medical Sciences, Kermanshah, Iran; 5 School of Public Health, Tehran University of Medical Sciences, Tehran, Iran; 6 Ahvaz Jundishapur University of Medical Sciences, Ahvaz, Iran

**Keywords:** work engagement, staff, hospital, enthusiasm

## Abstract

**Objective:**

Work engagement is a new concept in the field of psychology and human resource management. Increased vitality and enthusiasm is a social phenomenon that brings work engagement for society. This study aimed to evaluate work engagement and its determinants in Kermanshah hospitals’ staff.

**Methods:**

This cross-sectional study was conducted on 387 hospital administrative, clinical, paraclinical, and service staff. The sample size was calculated using Krejcei-Morgan table. The data were collected using a questionnaire including demographic characteristics and job engagement components. Then, the data were analyzed using descriptive statistics as well as independent sample t-test and one-way ANOVA.

**Results:**

The participants’ mean (SD) of age was 32.63±2.7 years and most of them were female (57.6%). The results revealed a significant relationship between work engagement and age group (P=0.01) and work experience (P=0.04). However, no significant relationship was found between work engagement and sex, education level, and job unit.

**Conclusion:**

The results of this study showed that only job experience and age were associated with work engagement. However, no significant relationship was found between work engagement and education level, sex, and job. Thus, further studies are suggested to investigate the cultural factors and personality traits associated with job enthusiasm among the hospital staff, especially nurses.

## 1. Introduction

Organizations are an important part of many individuals’ lives. Businesses are organized and people with membership in organizations spend many hours of their life in these places. If individuals work in an organization with enthusiasm and passion and are dependent on their job, the organization will be successful in achieving its objectives, and the community will also benefit from higher vitality (Gill, 2008). Nowadays, work engagement has received increasing attention by both practitioners and academicians (Mirza Darani, 2013). Work engagement is a new concept in the field of psychology and human resource management. This concept is defined as a positive, fulfilling, work-related state of mind that is characterized by vigor, dedication, and absorption (Schaufeli et al., 2002). Increased vitality and enthusiasm is a social phenomenon that brings work engagement for society (Poorabbas, 2008).

Work engagement has a positive relationship with outcomes such as job performance, organizational citizenship behaviors, and job satisfaction, but a high negative correlation with turnover intention. Work engagement has three dimensions, namely attraction, motivation, and devotion to force. In the attraction dimension, an individual is involved at working hard; such a way that it provides one with a very enjoyable experience and refers one to concentration on and immersion in his work. This makes it difficult for individuals to withdraw from their job (Schaufeli et al., 2006). In the intension dimension, an employee shows considerable efforts in order to do one’s job and is more persevere in difficult situations. Most capable employees are motivated by their work and show more resistance in case of being faced with interpersonal conflicts (Schaufeli et al., 2002). The third dimension of work engagement is devotion that is marked by severe psychological conflicts and it is a combination of sense of importance, enthusiasm, and challenges (Noami & Piriaei, 2011).

If work engagement runs incorrect and incomplete, cynicism and doubt will affect the whole organization (Asgari Bajgarani, 2011). On the other hand, the positive effects of job enthusiasm are beneficial for both organizations and individual staff. These benefits contribute to employee productivity and higher wages, increase the staff’s self-esteem, and improve the workers’ health (Saks, 2006). Job motivation is a variable leading to direction, intensity, and persistence of occupational behavior (Kanfer & Ackerman, 2000). The quality of the relationships between managers and employees, manager’s trust, and individuals’ confidence in their desire to work can enhance the employees’ passion (Chughtai & Buckley, 2011). Managers need to identify the key components of work engagement and design methods to assess staff development in this field.

According to Hayase’s perspective, the components of work engagement include enthusiasm, diligence, and immersion at work (Hayase, 2009). Enthusiasm at work: energy, flexibility, and mental vitality are critical elements in competitions and challenges.

Diligence at work is described as perseverance, seriousness, and passion at work. Assiduous employees insist on doing things in the right way when faced with problems. Drowning at work is a type of conceptual conflict that involves sustained attention and such employees are extremely focused on their tasks and lose their consciousness to the environment.

Work engagement is a widespread and relatively new concept in the field of occupational psychology and human resource management. Attention to work engagement has its roots in the studies conducted on job burnout (Nouri et al., 2011). Work engagement is a new paradigm in the field of human resources and because of its various usages, it has multiple definitions (MacLeod & Clarke, 2009). Work engagement fully mediates the impact of job resources on proactive behaviors at work; i.e., an increase in job resources is related to an increase in work engagement that is positively related to proactive work behaviors (Salanova & Schaufeli, 2008).

A study conducted in 2011 showed that organizational culture had a significant positive, indirect relationship with occupational motivation. This means that the cultural working groups are crucial, jobs provide sufficient information for the staff, there are good relationships among employees, all personnel are involved in the decision makings which are related to their job, management and supervisors will provide clear feedback to subordinates, and the emotional impacts on employees increase their motivation (Noami et al., 2012). However, lack of enthusiasm in health professions can not only put the patients’ lives at risk, but can also cause the costs of equipment and hospitals to be wasted. In addition, it may lead to demotivation in other employees. Given the importance of work engagement and its role in individual and organizational productivity, review and scrutiny of this variable and identification of the effective factors are required. Considering the hospital staff’s contacts with patients, hospital staff burnout, and sensitive conditions of hospitals, the present study aims to evaluate work engagement and its determinants in Kermanshah hospitals’ staff.

## 2. Methods

This cross-sectional study was conducted on 387 hospital administrative, clinical, paraclinical, and service staff. The sample size was calculated using Krejcei – Morgan table. The samples were selected from Kermanshah hospitals’ staff using simple random sampling. The data were collected using a questionnaire including demographic characteristics and job engagement components. The first section of the questionnaire included questions on demographic characteristics, such as age, sex, education level, occupation, and place of work. The second section of the questionnaire consisted of 17 questions related to work engagement. The components of work engagements were diligence, enthusiasm, and immersion in job (Schaufeli et al., 2006).

The questionnaire scores were categorized as follows: high to very high within the range of >75 percentile, average work engagement within the range of 25 to 75 percentile, and very low to low within the range of <25 percentile. The content validity of the questionnaire was evaluated and confirmed by experts. In addition, its reliability was calculated using Cronbach’s alpha test. Cronbach’s alpha reliability coefficient ranges from zero to 1 and the closer this number is to ^+^1, the more reliable the questionnaire is. Cronbach’s alpha for the work engagement questionnaire was obtained as 0.75, which indicates the reliability of the questionnaire. The questionnaire was distributed among the hospital staff. The inclusion criteria of the study were signing written informed consents, not suffering from mental problems, and having worked in that workplace for more than a year. The individuals who did not meet these criteria were excluded from the study. After all, the data were entered into the SPSS statistical software and analyzed using descriptive statistics. Besides, independent sample t-test and one-way ANOVA were used to analyze the differences between and among groups, respectively.

## 3. Results

The participants’ mean (SD) of age was 32.63±2.7 years and most of them were female (57.6%). The most and least frequent age groups were over 35 years and 25-20 years, respectively. Besides, the participants’ education levels ranged from high school diploma to master’s degree, with bachelor’s degree being the most common one (53.7%). In addition, 30% of the study participants had over 15 years of working experience (Table 1).

**Table 1:** Distribution of subjects according to work experience

Work Experience (Year)	Frequency	Percent
< 5	106	28.2
6-10	99	25.6
11-15	63	16.3
> 15	116	30
100	387	total

The highest number of the study participants was related to the emergency department (35.7%), while the lowest number was from the treatment section (0.8%) (Table 2).

**Table 2:** Distribution of subjects according to job units

Job units	Frequency	Percent
Radiology	35	9
Pharmacy	22	5.7
Physiotherapy	10	2.6
Emergency	138	35.7
Maternity	57	14.7
Administrative	122	31.5
treatment	3	0.8
total	387	100

Moreover, 78.8% of the personnel identified with their jobs, but 21.2% had failed to establish proper communication with their job and were not satisfied. Job units were compared in terms of career passion using t-test. The results revealed no significant differences between the job units. Additionally, the scores of work engagement were moderate in all the units and none of the subjects had a top score of work engagement (Table 3).

**Table 3:** Comparison of work engagement scores in different job units

	Job units	Frequency (%)	Mean	SD	P-Value
work engagement	Emergency	138(35.65)	52.19	10.84	0.53
	Maternity	57(14.73)	53.01	7.73	
	Administrative	122(31.52)	53.92	8.71	
	other	70(18.09)	52.55	8.71	

Work engagement was also compared among age groups and the results demonstrated a significant difference among the age groups in this regard (Tables 4 and 5).

**Table 4:** Comparisons of work engagement scores in age groups

	Age groups	Frequency (%)	mean	SD
work engagement	20-25	57(14.73)	49.73	9.78
	26-30	17(30.23)	51.34	9.16
	31-36	88(22.74)	55.17	55.17
	More than 35	125(32.29)	54.17	54.29

**Table 5:** Work engagement compared between age groups

Age group		The absolute mean difference	P-Value
20-25	31-35	5.44	0.00
20-25	More than35	4.55	0.01
26-30	31-35	3.83	0.02

work engagement was compared in groups with different work experience and it was shown that engagement was significantly different between people with more than 15 years’ work experience and people with less than 5 years’ work experience (P= 0.04) (Table 6).

**Table 6:** Work engagement compared between work experience groups

	Work experience groups (year)	Frequency (%)	Mean	SD
work engagement	< 5	109(28.2)	50.8	8.91
	6-10	99(25.6)	53.28	9.85
	11-15	63(16.3)	53.63	12.33
	>15	116(30)	54.22	7.88

Work engagement was also compared among the groups with different educational degrees and the findings indicated that educational degree had no significant effects on job enthusiasm (Table 7).

**Table 7:** Work engagement compared based on education degrees

P-Value	Education level	Percent (%)	Mean	SD	P-Value
work engagement	diploma	68(17.6)	54	10.86	0.4
	Associate degree	48(12.4)	53.81	10.37	
	bachelor	208(53.7)	52.83	9.46	
	MSc	63(16.3)	51.38	7.61	

In this study, no significant difference was found between the participants who communicated and those who did not communicate with their job concerning the mean score of work engagement (P=0.58). The mean score of work engagement was 52.92 and 52.93 in females and males, respectively but the difference was not statistically significant (P=0.99).

The total mean score of work engagement regardless of its components was 50.5±8.9. Based on the general grouping of the questionnaire, this value was in the moderate group. In addition, the mean scores of enthusiasm at work and diligence at work were 10.5 and 25.3, respectively. These values were both moderate according to the components’ grouping scores. Finally, the mean score of drowning at work based on the number of questions was 14.7 which belonged to the weak group.

## 4. Discussion

The results of this study which was conducted on 387 Kermanshah hospitals’ staff showed that job experience and age were associated with work engagement. However, no significant relationship was found between work engagement and education level, sex, and job units. The results also demonstrated that the participants within the age range of 20-30 years had less enthusiasm for job.

Besides, work engagement scores were higher in those who had higher working experience. With increase in experience, personality will be more stable, the ability to adapt and cope with the problems increases, and thus passion will be greater. Similar results were also obtained in the study by David Gill (Gill, 2008).

Two other studies were also conducted in nurses of Ardebil and Ahwaz universities of medical sciences. In these studies, managers’ leadership style and organizational learning had an impact on job enthusiasm (Naghizadeh & Baghi, 2013; Allipourbirgani, 2013). Moreover, two other studies revealed a significant relationship between work engagement components and organizational commitment, and that the participants who had more job passion had greater job commitment (Hayase, 2009). Another study conducted among nurses showed that with the increase in workplace stress, job enthusiasm was reduced (Mei-Ling, 2010). In our study also, hospital employees had moderate scores of work engagement, and high passion scores were not observed in job units. This result might be due to the stress in the hospital units, which reduces the work engagement score.

In several studies, including a study conducted in Taiwan, which investigated the factors affecting the nurses’ work engagement, some factors related to work engagement were revealed, including personality, organizational factors, and cultural factors (Mei-Ling, 2010; Hakanen & Lindbohm, 2008). Moreover, the study conducted by Auli and Hakann showed that occupational capability affected work engagement and employment compatibility related to individuals’ work experience, physical condition, smoking, and alcohol abuse (Auli et al., 2012). The results of our study also demonstrated that work experience had an impact on work engagement.

A previous study which was conducted in industrial organizations showed that women’s mean work engagement was 63.09, while this measure was 52.9 in the present study (Shokrkon et al., 2008). The difference between these two results could be attributed to the differences in the work environment and job conditions. Moreover, existence of stress in the hospital environment could reduce job enthusiasm. Thus, the recommendation of Freeney and Tiernan regarding provision of facilities for hospital staff can also be applied in our hospitals (Freeney & Tiernan, 2009).

In addition to examining work engagement in hospital, Duffy and Lim assessed and compared work engagement in other places, such as homes and sports centers (Lim et al., 2010; Duffy et al., 2011). However, this was not performed in our study, which is one of the limitations of the investigation. Another study limitation was that the effects of some variables, such as job satisfaction and job management, were not considered.

## 5. Conclusion

In this study, all the participants had moderate work engagement scores; therefore, managers must increase the staff’s job satisfaction. However, some organizations do not take this issue into account. We can help the staff to enhance work engagement by applying creative and innovative plans as well as by timely staff encouragement and support. Understanding job status can also affect work engagement. Hospital’s reputation and validation can in turn be enhanced through work engagement. Given that work engagement was higher in the experienced staff compared to others, job satisfaction should be considered for the less experienced staff. The findings of this study can provide the basis for further researches. Future studies are suggested to investigate the cultural factors and personality traits associated with job enthusiasm among the hospital staff, especially nurses.
